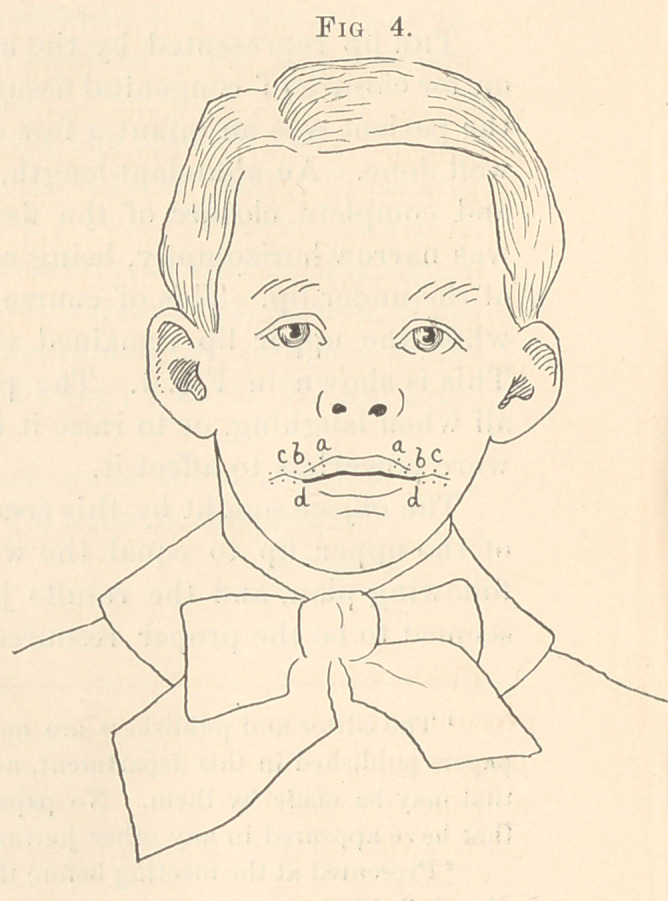# Secondary Operation for Harelip

**Published:** 1898-09

**Authors:** Thomas Fillebrown

**Affiliations:** Boston


### International Dental Journal.

VOL. XIX. SEPTEMBER, 1898. NO. 9.

### Original Communications.[1]

### SECONDARY OPERATION FOR HARELIP.[2]

BY DR. THOMAS FILLEBROWN, BOSTON.

THE lip represented by the accompanying figures was operated on for closure of congenital fissure by the attending physician when the patient was an infant a few days old. The operation was very well done. An abundant length, vertically, of the lip was obtained, and complete closure of the fissure. Of necessity the upper lip was narrow horizontally, being not more than two-thirds the width of the under lip. This of course made the under lip pout forward, while the upper lip remained very flat, receding, and immovable. This is shown in Fig. 1. The patient was unable to widen it at all when laughing, or to raise it under any condition. The muscles were powerless to affect it.

The object sought by this secondary operation was the widening of the upper lip to equal the width of the under. I adopted the following plan, and the results justified my selection. The cheeks seemed to be the proper resource, and I proceeded as follows: I

[1] The editor and publishers are not responsible for the views of authors of papers published in this department, nor for any claim to novelty, or otherwise, that may be made by them. No papers will be received for this department that have appeared in any other journal published in the country.

[2] Presented at the meeting before the American Academy of Dental Science, March 2, 1898.

Fig. 1.

Fig. 2.

Fig. 3.

Fig 4.

made the incisions *a b*, *a b*, Fig. 3, from the corners of the mouth backward a little more than half way through the cheeks. I then cut out and dissected off oblong flaps *e e*, Fig. 3, from the inside of the mouth, represented by the dotted lines, Fig. 3.

The parts were now free from each other, and I drew the flaps of mucous membrane through the incision and sutured them to the edge of the skin on the outside of the incision.

This made a red border for the additions to the lip. To gain the additional width I made a perpendicular incision from *b* directly upward one-half the length of the incision, *a b*. Then I straightened out the last incision, bringing the point *a*, Fig. 3, to *a*, Fig. 4, the point *b*, Fig. 3, to *b*, Fig. 4, and *c*, Fig. 3, to *c*, Fig. 4. The sutures on each side held the edges together on the outside and two within the mouth closed the gap left by the taking of the flap for the red border of the lip.

This carried all the new red border into apposition with the red border of the under lip and made them of equal width. The result is shown in Fig. 2. The size of his mouth was increased one-half. In two months the boy could show all of his upper teeth when laughing, and could give as good and hearty a whistle as any boy of his age, an accomplishment of which he was very proud.

The right nostril was considerably flattened and too large. A V-shaped incision, as represented in Fig. 3, corrected this feature and made the nares almost symmetrical. Of course, there was a slight fault in the orbicularis muscle at the corners of the mouth, but that almost wholly disappeared in the course of six months.

The general plan of the incision used in this case can be applied with advantage in many cases where sufficient tissue and mobility are not obtained at the first operation.

## Figures and Tables

**Fig. 1. f1:**
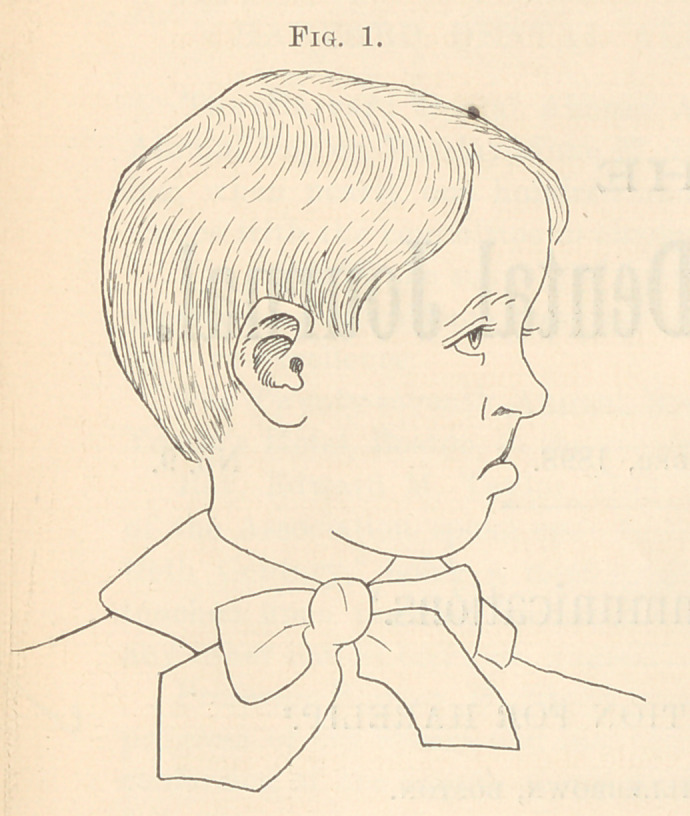


**Fig. 2. f2:**
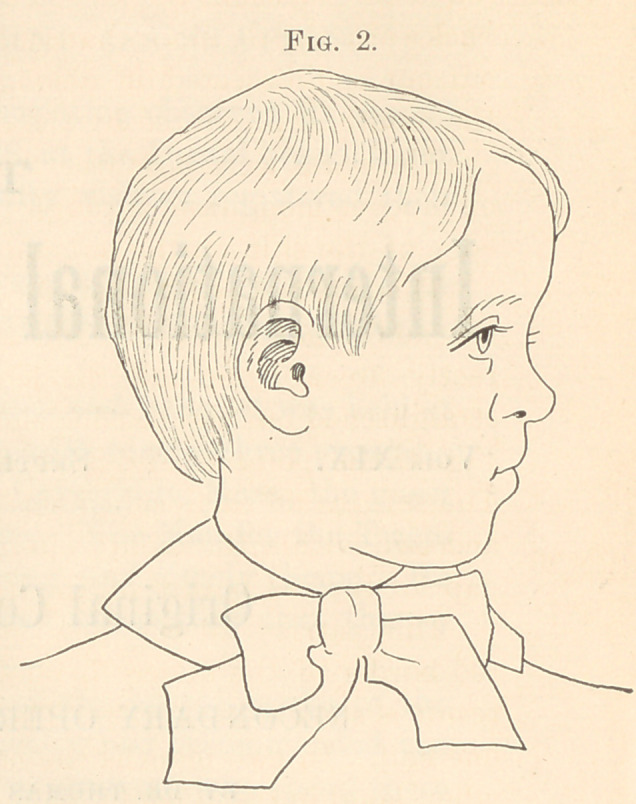


**Fig. 3. f3:**
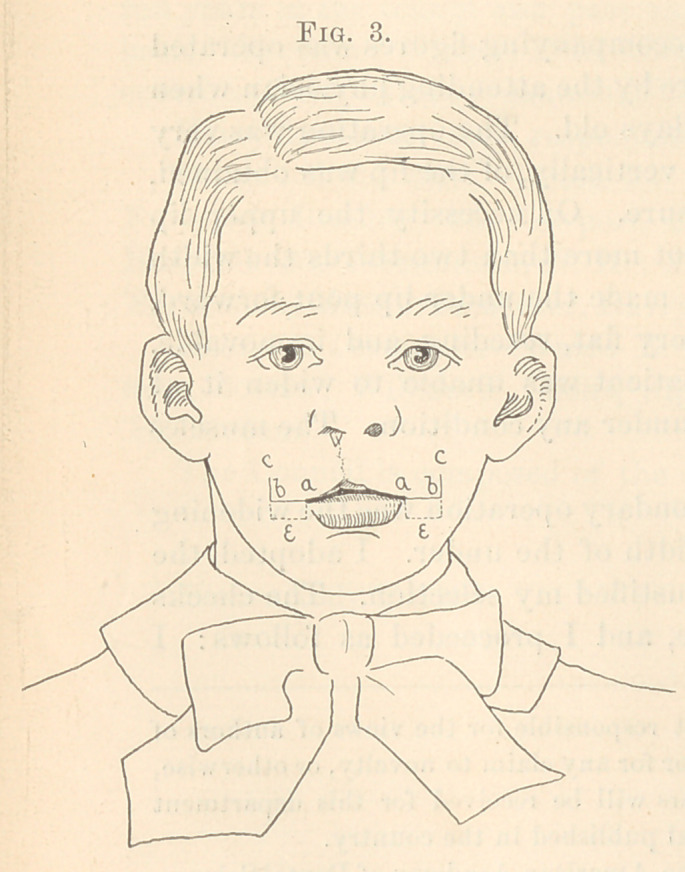


**Fig 4. f4:**